# Diagnostic radiographer advanced clinical practice in the United Kingdom – A national cross-sectional survey

**DOI:** 10.1259/bjro.20210003

**Published:** 2021-06-24

**Authors:** Nick Woznitza, Lisa Pittock, James Elliott, Bev Snaith

**Affiliations:** 1 Department of Radiology, University College London Hospitals, London, United Kingdom; 2 Department of Radiology, Homerton University Hospital, London, United Kingdom; 3 School of Allied & Public Health Professions, Canterbury Christ Church University, Canterbury, United Kingdom; 4 Maidstone Nuclear Medicine Department, Maidstone and Tunbridge Wells NHS Trust, Royal Tunbridge Wells, United Kingdom; 5 Radiology, Mid Yorkshire Hospitals NHS Trust, Dewsbury, United Kingdom; 6 Faculty of Health Studies, University of Bradford, Bradford, United Kingdom

## Abstract

**Objectives:**

To survey the diagnostic radiography workforce in the United Kingdom (UK) at an organisational level to ascertain the scope of advanced practice and compliance with Health Education England standards for multiprofessional advanced clinical practice (ACP).

**Methods:**

174 diagnostic imaging departments were invited to participate in a cross-sectional electronic survey focused upon advanced level practice and their educational and accreditation expectations (October–December 2019). Breast imaging, computed tomography, fluoroscopy, interventional radiology, lithotripsy, magnetic resonance imaging and projectional radiography were included.

**Results:**

A total of 97 responses were received, of which 79 were eligible for inclusion (45%). Respondents reported advanced-level practice roles across all imaging modalities, which included clinical reporting, procedural-based and combined roles. Radiograph and mammogram reporting were most prevalent (95 and 67% of Trusts), with fluoroscopy the most frequent procedure-only role (25%). Only 39% of trusts required adherence to the four pillars of ACP within job descriptions, and only 12% requiring a full Masters qualification.

**Conclusions:**

Diagnostic radiographer reporting and procedure-based roles in the NHS are varied and widespread. However, inconsistencies in fulfilment against the expected standards for advanced practice exist. Realignment of advanced-level roles to delineate enhanced and advanced clinical practice may ensure consistency between roles and professions. A requirement for accreditation as an advanced (clinical) practitioner with adherence to advanced practice requirements could therefore provide value to accreditation for both individual practitioners and Trusts.

**Advances in knowledge:**

Within the UK, diagnostic radiographer roles previously self-identified as advanced-level practice may be termed enhanced practice when not adhering to expected ACP standards.

## Introduction

Diagnostic radiography has a long history of role development^1^; however, the concept of advanced practice within the United Kingdom (UK) was formally introduced as part of the 2003 Radiography Skills Mix report.^2^ Subsequent UK education^3,4^ and career frameworks^5,6^ have been developed at advanced and consultant levels, as well as an assistant practitioner tier to grow the diagnostic imaging workforce.^2,7^ Advanced radiographic practice requires individuals to operate at an expert clinical level, but it also expects a range of different skills and capabilities across three further ‘‘pillars” mirroring the consultant practice functions^8,9^ including leadership and management; education and training; research, audit and service evaluation.

In 2017, Health Education England (HEE) launched a Multi professional Framework for Advanced Clinical Practice^10^ (ACP) and the other home countries have introduced similar expectations. The aim of these frameworks is to standardise the level of advanced practice across the non-medical workforce and provide assurance of the capabilities and competence of individuals. Similar to the HEE framework, in Wales, expectations for advanced practice are based on the four pillars and are laid out in the NHS Wales Post Registration Framework.^11^ However, in Scotland, there is evidence of poorer implementation of advanced practice due to multiple factors.^12^ Although better established in nursing, NHS Education for Scotland has committed to develop its advanced practice toolkit across the allied health professions.^13^ Previous research has highlighted discordance between advanced and extended practice, described as supplementary skills and responsibilities that extend beyond the statutory responsibilities and competencies at the point of professional registration.^14^ The distinction between advanced and extended or enhanced practice remains unclear, with the terms often used interchangeably both in research and clinical practice.^15–17^ This disparity is also evident in other healthcare professions.^18^ Many advanced practice roles have evolved over many years to incorporate a range of clinical settings. These roles developed without a set standard, resulting in inconsistency in expectation for qualifications, titles and level of practice.^19^

The evolution of radiographer advanced practice within the UK has been both organic and reactive to the service demands and clinician staffing within the National Health Service (NHS).^20^ International efforts in radiographer advanced practice are less established, although countries including Canada, Australia and New Zealand continue to explore implementation using a pre-defined framework.^21,22^ The most common area of diagnostic radiographer advanced practice has been the clinical reporting of imaging investigations as part of a multiprofessional team.^23–27^ Perhaps, less visible are advanced practice roles that incorporate a procedural clinical element such as administration of pharmaceuticals during nuclear medicine and CT cardiac stress examinations.^17,28,29^ To date there has not been a comprehensive review of the range of advanced practice radiographer roles within the UK workforce or how closely aligned radiographer roles are to the four pillars of advanced practice.

The aim of this study was to survey the UK diagnostic radiography workforce at an organisational level to establish the scope of advanced practice in diagnostic radiography and explore correlations between reported and expected standards for advanced clinical practice.

## Methods

### Cross-sectional survey

A cross-sectional electronic organisational survey of diagnostic imaging departments was undertaken, which focused on advanced level practice (October-December 2019). The questionnaire was created using onlinesurveys.ac.uk (Jisc, Bristol, United Kingdom) primarily constituted closed multiple answer questions exploring imaging modalities, concluding with in optional free-text response (see supplementary file). An invitation including a webpage link to the online survey was distributed to every UK NHS Trust via the imaging department manager using paper dissemination. The survey was additionally publicised by the Society and College of Radiographers (SCoR; UK) and the researchers’ professional networks (snowball sampling). The focus of the survey was advanced clinical practice in radiography. Sonography and nuclear medicine were considered outside of the scope of this work due to the multiprofessional nature of practice in these areas. The survey remained open for eight weeks after the initial invitation letter was distributed, with a reminder letter circulated to non-responding organisations at five weeks. Due to the parallel survey routes of paper and online survey, any duplicate responses from the same workplace were combined. Data were extracted to Microsoft Excel (USA, 2017) and descriptive statistics calculated. Institutional ethical approval was obtained prior to data collection (University of Bradford EC25681).

## Results

All acute and specialist NHS Trusts in England (*n* = 152), and Health Boards in Scotland (*n* = 14), Wales (*n* = 3) and Northern Ireland (*n* = 5) were invited to participate. Of the possible 174 responses, a total of 97 responses (paper webpage link *n* = 54, 56%; online webpage link *n* = 43, 44%) were received, of which five were ineligible and 13 were duplicates from the same NHS Trust, resulting in 79 responses (of 174, 45%) included within the analysis. Responses were predominantly from England (*n* = 69 of 152, 45%), although Northern Ireland (*n* = 2 of 5, 40%), Scotland (6 of 14, 43%) and Wales (2 of 3, 67%) were also represented. The employment of radiographers to undertake advanced level practice was reported at 95% of responding Trusts (*n* = 75) across a wide range of imaging modalities; breast imaging, CT, projectional radiography, fluoroscopy, interventional radiology, lithotripsy and magnetic resonance imaging (MRI). Four Trusts (5%) indicated that they had no diagnostic radiographer advanced practice roles and do not have plans to implement them.

Most respondents indicated advanced practice roles within multiple modalities in the same Trust, although not one Trust had roles across all seven modalities. Four specialist or tertiary Trusts do not utilise radiographer radiograph reporting.Table 1 provides a breakdown of reporting and procedural elements for each modality.

**Table 1.** Radiographer advanced practice within different imaging modalities

Modality	Reporting only	Procedural only	Combined roles	Not occurring within Trust
Breast imaging	6	1	49	23
Computed tomography	27	3	4	45
Projectional radiography	61	0	10	8
Fluoroscopy	0	15	36	28
Interventional radiology	0	0	22	57
Lithotripsy	0	5	1	73
Magnetic resonance imaging	27	3	0	49

### Advanced radiographic practice across trusts

Reporting and procedural (non-reporting) roles were found in isolation or in combination across many Trusts. Radiograph and mammogram reporting were most common, occurring in 71 (95%) and 50 (67%), respectively. Cross-sectional reporting was less frequent, occurring in 31 (41%) and 27 (36%) of Trusts for CT and MRI.

As a modality, projectional radiography exhibited the fewest procedural advanced practice roles (*n* = 10, 13%), of which radiographer-led/assisted discharge from emergency care (*n* = 6) were the majority. The remaining four indicated ‘other’ advanced roles but did not provide further detail. Breast imaging advanced practice roles were variable with combined (*n* = 49, 89%), procedural only (*n* = 7, 12%) and reporting only (*n* = 5, 9%) instances across 55 Trusts. Procedural roles within breast imaging included ultrasound, ultrasound-guided interventions and stereotactic biopsies. Fluoroscopy had the greatest procedural-only roles with 13 (of 51, 25%) Trusts independently performing examinations without providing the clinical report. There were no examples of reporting-only advanced practice roles in fluoroscopy, lithotripsy or interventional radiology. Line insertion was the most common area of interventional radiology advanced practice, occurring as the only element (*n* = 6) or in combination with other interventional procedures in 16 of the 22 Trusts. Other interventional procedures performed by diagnostic radiographers included nasogastric tube insertion, diagnostic angiography, hysterosalpingograms, assisting with radiofrequency ablation and tube checks.

Imaging modalities with fewer instances of procedural advanced practice roles include CT (7 of 34, 21%), lithotripsy (6 of 6, 100%) and MRI (3 of 30, 10%). Activities in CT colonography and angiography involved administration of medication under a patient group directive at three Trusts, with four trusts working in combination with reporting roles. Lithotripsy roles included pre-treatment planning (*n* = 6) and outpatient follow-up clinics (*n* = 2). One Trust indicated pharmacological stress imaging for cardiac patients in MRI.

### Requirements for advanced clinical practice across Trusts

There was significant variability in the requirement for all four pillars of advanced clinical practice to be included within diagnostic radiographer-advanced practice rolesTable 2.

**Table 2.** Requirements for advanced clinical practice across Trusts

Four pillars of Advanced Clinical Practice	MSc required	MSc encouraged	No requirement	Total
Expected in role, within job description	3	18	8	29
Expected in role, not explicit in job description	0	13	9	22
Stated in job description but not managed across all pillars	3	5	3	11
No expectation	1	1	5	7
Different expectations depending on modality	2	2	2	6
Total	9	39	27	75

Most Trusts (*n* = 51, 68%) expected diagnostic radiographer advanced practitioners to be working across all four pillars as defined by HEE and the other home countries, but only 39% (*n* = 29) included this within advanced practice job descriptions. Seven Trusts required diagnostic radiographer-advanced practitioners only to fulfil the expert practice pillar of the HEE ACP framework.^1^ Few Trusts (*n* = 9, 12%) required diagnostic radiographers to complete an MSc although this was encouraged in the majority of Trusts (*n* = 39, 52%). One response indicated that they promoted the MSc qualification amongst staff but there was little interest.Table 3

**Table 3.** Trust requirement for CoR accreditation as an ACP and to fulfil all 4 pillars of advanced clinical practice

Four pillars of advanced clinical practice	CoR accreditation as an advanced practitioner required			Total
	Yes	No	Unsure	
Expected in role, within job description	5	4	20	29
Expected in role not explicit in job description	5	9	8	22
Stated in job description but not managed across all pillars	2	6	3	11
No expectation	0	5	2	7
Different expectations depending on modality	1	4	1	6
Total	13	28	34	75

CoR, College of Radiographers.

Free-text responses suggested that not enough value is perceived in CoR accreditation relative to the time taken *“We would like our Advanced Practitioners to be accredited with the SCoR but have not had the time to pursue this.”*

In the UK, all non-medical healthcare staff, including diagnostic radiographers, are employed using standard terms and conditions of service, known as Agenda for Change. Pay bands (Band 2–9) within agenda for change are determined by matching job roles to agreed job profiles, based on responsibilities of the post and determine financial reimbursement. The ranges of Agenda for Change pay bands for radiographer advanced practitioners are shown inTable 4 .

**Table 4.** Distribution of Agenda for Change pay bands

Agenda for Change Pay Band	Number of Trusts*
Band 6	14
Band 7	71
Band 8a	29
Band 8b	7
Band 8c	0
Band 8d	1

a Some Trusts reported multiple pay levels and therefore the total exceeds the number of respondents

Radiographers in advanced practice roles were predominantly employed at band 7 and 8a, with 35 Trusts consistently implementing advanced practice roles at band 7 across different imaging modalities. Band 6 roles were frequently identified as training posts (*n* = 8), although some at this level were performing, but not reporting, fluoroscopy examinations (*n* = 4) or undertaking a single area of breast advanced practice (*n* = 2). Free-text data on the differences between bandings identified that in some instances those in higher banded roles (8a+) also had combined leadership responsibilities within the department and oversight of service delivery, although this was not universal as some sites specified that 8a roles were offered for areas considered to represent specialist reporting for example, CT lung nodules, fluoroscopy or paediatrics. One Trust indicated that the job description and expectations for the Band 7 and Band 8a reporting radiographer were identical.

## Discussion

Using information from service leads in radiography across the UK, this research has identified that advanced practice by diagnostic radiographers is widespread across the NHS, with diverse roles within all imaging modalities, including clinical reporting and procedure-based practice. The expectations of individual roles and resultant practice are inconsistent across the UK, with varying engagement with the four pillars of advanced practice. The spectrum of advanced practice roles appears to have been developed organically in response to changing service needs over time, an evolution rather than centrally led transformation.

The evolution of advanced practice has intensified in the UK over the last two decades, and the national frameworks have established new standards which may be at odds with current service delivery models and practitioner roles.^10,11,13^ Importantly, these build on the professional body (SCoR) requirements^5^ and expect individuals to transition to a unified definition of advanced clinical practice and thus aim for greater standardisation of the non-clinical expert pillars. Variation in expectations of practitioners to fulfil each of the four pillars was found, maybe pointing to a lack of understanding at an employer as well as individual practitioner level. The current results mirror the recent work of Harris et al^30^ who found that many job descriptions for advanced radiographer roles met professional body standards but differed from the HEE ACP framework.^10^ This would confirm that there are many radiographers with ACP-level expert clinical practice but who are working at an enhanced level when the wider advanced clinical practitioner capabilities are considered.

A contributing factor to the disparity between current practice and expectations could be the lack of Masters (Level 7) educational underpinning, with only 12% of Trusts requiring an MSc for advanced roles. In addition, some areas of procedure-based advanced practice may lack academic development due to the implementation novel practices and a resultant gap in education provision. This raises the question whether the definition of Masters level is being considered by many to represent any postgraduate academic award, rather than a full degree or the equivalent of this by experiential learning. This mis-match requirement may be an ongoing barrier to diagnostic radiographer ACP roll out or transferability of practitioners between Trusts as education in (and assessment against) the non-clinical pillars may be limited. It also implies that progression to complete a doctorate (or experiential equivalent) may also be limited if Masters level education is not sufficiently valued and therefore will reduce opportunities for advanced practitioners to progress to consultant roles.^31^

There has been a shift in radiographer practice over the last 20 years; some tasks previously considered to be ‘advanced’ are now performed by practitioner radiographers, for example, the administration of intravenous contrast. Price et al found fluoroscopy, specifically performing barium enemas, was the most frequent extended role occurring in 119 of 172 Trusts (69%).^1^ While a similar proportion (51 of 75, 68%) have radiographer advanced practice in fluoroscopy in the current study, the majority (36 of 51, 71%) combine performing the examination with the provision of the clinical report, increasing from the 20% previously^1^ (34 of 119 Trusts). One consequence of practice evolution could involve the transition of unidimensional “advanced” radiographer practice that focuses solely on expert clinical elements. For example, clinical reporting or performing fluoroscopy procedures could now be classed as extended or enhanced practice, and that to be considered ACP it would require all four pillars and possibly registration or accreditation by the CoR, HEE or the Health and Care Professions Council. Within England, the emerging HEE Centre for Advancing Practice could play a role in standardising the requirement for non-expert practice elements but radiographers would need to demonstrate value and impact to Trusts and practitioners, particularly to justify funding for the full MSc award.

To date, there has been little uptake for accreditation as an advanced practitioner by diagnostic radiographers, perhaps influenced by a lack of awareness of employers, as only 6% (*n* = 5) of responding Trusts in our survey require CoR accreditation for advanced practice. There does not appear to be any link between an expectation that all four pillars of advanced practice are fulfilled by radiographers and employer expectation for individual accreditation as an advanced practitioner. The results of this study echo the recent work by Deane et al with little perceived value in accreditation, although this was at an individual practitioner level.^32^ It will be vital that the benefits of accreditation as an ACP are communicated effectively to employers, existing and future practitioners.

Most advanced practice roles were found to be banded under the Agenda for Change (AfC) criteria at primary band 7 and to a lesser extent 8a. Some higher banding was indicated to be based on a desire to attract staff. Inconsistencies existed, including a case within a single Trust where job responsibilities were the same for differently banded posts. Many of the higher banded roles expect leadership or management responsibilities. It raises the question whether advanced practice that meets all four pillars should be banded at 8a or above and the highly specialist/enhanced roles in reporting and procedure-based practice that do not meet all pillars should be band 7. This would ensure consistency between professional groups whilst recognising the important practice of these roles at all levels.

This study provides clear evidence of the widespread use of radiographers to undertake independent reporting of images across a range of different imaging modalities and to perform roles in procedure-based practice. Radiographer reporting is now an embedded feature of the imaging landscape along with a range of other activities. However, no defined structure has been identified for the implementation of such roles and the *ad hoc* evolution over several decades has likely had a major influence on the acknowledgement of, and expectations for, delivery at a higher level of practice. The lack of educational expectation and wider role responsibilities is appearing to inhibit the utilisation of radiographers to inform, lead and innovate service models, but rather focusing their time on clinical tasks. It is clear that ‘reporting radiographers’ and others undertaking complex elements of the clinical pathway in diagnostic imaging can positively impact service delivery, with economic benefits and opportunities for role progression.^20^

Limitations of this study includes the exclusion of multiprofessional imaging modalities such as nuclear medicine and ultrasound. Furthermore, Trusts were asked to self-identify advanced practice and were not asked whether all imaging modalities were within their Trust.

## Conclusion

Diagnostic radiographer advanced practice roles, both clinical reporting and procedure-based practice, are widespread and embedded across the NHS. The variety of roles is significant and address local specialist service needs. However, inconsistent fulfilment of all pillars of ACP, lack of Masters education and evolution of practice could see some ‘advanced practitioner’ radiographers more aligned with enhanced practitioners. The important contribution of such roles, in terms of service delivery, should not be underestimated and are key to radiographer career progression and the sustainability of imaging capacity. It is recognised that advanced clinical practice implementation may have changed due to the effects of the COVID-19 pandemic on NHS working practices. Future studies could map advanced practice before and following the pandemic. Accreditation at the advanced practice level could help ensure consistency and parity of roles both between and within Trusts and across professions, but the value of accreditation needs to be recognised for both individual practitioners and employers.
